# Studying rhythm processing in speech through the lens of auditory-motor synchronization

**DOI:** 10.3389/fnins.2023.1146298

**Published:** 2023-03-02

**Authors:** Lu Luo, Lingxi Lu

**Affiliations:** ^1^School of Psychology, Beijing Sport University, Beijing, China; ^2^Laboratory of Sports Stress and Adaptation of General Administration of Sport, Beijing, China; ^3^Center for the Cognitive Science of Language, Beijing Language and Culture University, Beijing, China

**Keywords:** auditory-motor synchronization, rhythm, speech, finger-tapping task, speech-to-speech synchronization task

## Abstract

Continuous speech is organized into a hierarchy of rhythms. Accurate processing of this rhythmic hierarchy through the interactions of auditory and motor systems is fundamental to speech perception and production. In this mini-review, we aim to evaluate the implementation of behavioral auditory-motor synchronization paradigms when studying rhythm processing in speech. First, we present an overview of the classic finger-tapping paradigm and its application in revealing differences in auditory-motor synchronization between the typical and clinical populations. Next, we highlight key findings on rhythm hierarchy processing in speech and non-speech stimuli from finger-tapping studies. Following this, we discuss the potential caveats of the finger-tapping paradigm and propose the speech-speech synchronization (SSS) task as a promising tool for future studies. Overall, we seek to raise interest in developing new methods to shed light on the neural mechanisms of speech processing.

## 1. Introduction

Rhythm is a fundamental feature of human speech (Poeppel and Assaneo, 2020). As speech unfolds in time, it is organized into a hierarchy of quasi-rhythmic components along multiple timescales, such as phonemes, syllables, and prosody (Gross et al., 2013). Accurate processing of these hierarchical rhythmic structures lays the foundation of verbal communication.

Researchers have been tackling how the brain processes rhythms in speech for decades. The classic motor theory of speech perception put forward the interaction between auditory and motor systems as a promising solution (Liberman et al., 1967; Liberman and Mattingly, 1985; Galantucci et al., 2006). Accumulating empirical evidence has supported and extended this idea by showing bidirectional auditory-motor interactions in speech rhythm processing. Specifically, motor cortices track speech rhythms during passive listening (e.g., Wilson et al., 2004; Keitel et al., 2018). Meanwhile, speech production also recruits and shapes hearing (e.g., Houde and Jordan, 1998; Assaneo et al., 2021).

However, a critical question remains: How does the auditory-motor interaction work at different rhythmic structure levels? The few studies that addressed this issue revealed inconsistent findings. For example, some showed that motor areas could only track faster and lower-level speech rhythms, i.e., at the syllable level around 4 Hz (Ding et al., 2016; Sheng et al., 2019). Another study reported motor tracking of slower, higher-level rhythm, i.e., at the phrasal level (0.6–1.3 Hz) (Keitel et al., 2018). Moreover, all these studies focused on how auditory rhythms entrain the motor system. Evidence is lacking in the other direction: whether producing different speech rhythms affects hearing differently remains unknown. Therefore, it is time to seek new approaches to solving this question, especially those with behavioral tasks that require rhythm production.

Beyond the realm of speech processing, recent years have witnessed significant progress regarding the role of auditory-motor interaction in general rhythm processing. Many studies exploited a behavioral phenomenon known as auditory-motor synchronization, where participants synchronize their movements to external rhythms. Auditory-motor synchronization can come in many forms of movements, such as cycling limb movements and whole-body dancing (e.g., Chen H.-Y. et al., 2006; Toiviainen et al., 2010; Witek et al., 2017). The most commonly used paradigm is finger tapping, which has a long history (e.g., Fraisse, 1966; Michon, 1967; Keele et al., 1985) and remains popular to this date (for thorough reviews, see Repp, 2005; Repp and Su, 2013). The synchrony performance is assessed by the timing difference between the taps and sound onsets or the variability of inter-tapping time intervals (Repp and Su, 2013). By explicitly translating the rhythm processing results in the brain to behavioral outputs, finger-tapping tasks provide a critical window to the underlying mechanisms.

Converging evidence has demonstrated that auditory-motor synchronization not only reflects basic beat-level perception and action timing (Cannon and Patel, 2021) but also drives more complex forms of rhythm perception, including those involving hierarchical rhythms (Iversen and Balasubramaniam, 2016). Recently, interest has grown in using speech stimuli (Lidji et al., 2011; Falk and Dalla Bella, 2016; Falk et al., 2017; Rathcke et al., 2021; Kliger Amrani and Zion Golumbic, 2022; Wei et al., 2022). Similar to the findings with non-speech stimuli, participants can accurately translate perceived rhythms in the speech into finger-tapping movements. Nevertheless, auditory-motor synchronization paradigms have so far inspired relatively fewer studies on speech rhythms.

This mini-review aims to evaluate the potential implementation of auditory-motor synchronization paradigms in understanding the processing of hierarchical rhythms in speech. First, we summarize key findings relating finger-tapping performance to general speech/language skills. Second, we establish the feasibility of using finger-tapping performance to study the processing of hierarchical rhythms by reviewing studies using non-speech stimuli. Third, we describe current finger-tapping research exploring speech processing. Finally, we address the limitations of the commonly used behavioral paradigms and present a promising new tool.

## 2. Relating finger-tapping performance to speech processing

While fewer studies used finger-tapping tasks to address rhythm processing in speech, deficits in these tasks have repeatedly been demonstrated in clinical populations (for a thorough review, see Ladányi et al., 2020).

For example, poor finger-tapping performance is found in children with atypical speech/language development, including specific language impairment (Corriveau and Goswami, 2009; Zelaznik and Goffman, 2010; Cumming et al., 2015), speech production disorders (i.e., stuttering) (Falk et al., 2015; Redle et al., 2015), and reading disorders (i.e., dyslexia) (Wolff, 2002; Thomson and Goswami, 2008). Meanwhile, in typically developed children, finger-tapping performance could also predict their speech/language skills, with better synchronizers scoring higher in (pre-)literacy tests and presenting more precise neural encoding of acoustic temporal information (Woodruff Carr et al., 2014; Bonacina et al., 2021; Kertész and Honbolygó, 2021). Finger-tapping deficits have also been found in children and adults who stutter (Smits-Bandstra et al., 2006; Falk et al., 2015).

These results add to the growing literature on the importance of rhythm processing in speech (Hickok et al., 2011; Ladányi et al., 2020). Moreover, the linkage between finger-tapping performance and speech-related deficits indicates a common underlying mechanism, laying the foundation for finger-tapping tasks in speech studies.

## 3. Studying processing of hierarchical rhythms with finger-tapping to non-speech stimuli

Most finger-tapping studies have featured auditory-motor synchronization at the basic rhythmic (or “beat”) level. Specifically, the most common stimuli were isochronous rhythms (e.g., Aschersleben, 2002; Krause et al., 2010), and participants tapped their fingers to each pulse of the auditory rhythm. Fewer studies addressed rhythms with hierarchical structures, even less with speech rhythms. In this section, we focused on studies using non-speech stimuli and addressed their implications for speech studies.

### 3.1. Modulating tapping performance by rhythmic structure

One approach to demonstrate auditory-motor interaction in processing higher levels of rhythms is to assess whether varying the rhythmic structure impacts the synchronization performance. When using non-speech stimuli, hierarchical rhythms could be easily introduced by manipulating the acoustic features of the sound elements. Combining with brain imaging techniques, researchers have examined the neural basis of auditory-motor interactions and gained insights into related cognitions (Chen J. L. et al., 2006; Zatorre et al., 2007; Chen J. L. et al., 2008; Kung et al., 2013).

For example, Chen J. L. et al. (2006) introduced hierarchical rhythms to an isochronous tone sequence by placing an intensity accent on every third tone. The attenuation between the unaccented and accented tones was varied, creating different levels of rhythmic saliency. The participants were instructed to synchronize their tapping to each tone, that is, at the basic rhythmic level. Auditory and dorsal premotor cortices showed BOLD responses and functional connectivity covaried with the saliency change. Interestingly, as the saliency increased, accented tones evoked significantly longer taps compared to unaccented tones. Meanwhile, no such difference was found in control trials where the rhythmic structure was compromised.

The rhythm complexity of the stimuli was manipulated in another series of studies (Chen J. L. et al., 2008; Kung et al., 2013). Results showed that accurate timing of tapping (i.e., inter-tap intervals and time asynchrony between tap and sound onsets) depended on the successful resolution of the rhythmic structure and was subjected to musical training experience (Chen J. L. et al., 2008; Kung et al., 2013). The fact that tapping at the basic rhythmic level is affected by changes in the perceived higher-level rhythms indicates a flexible top-down mechanism in coordinating motor outputs with external sounds.

### 3.2. Interfering rhythm processing by active finger tapping

Since the auditory-motor interaction is bidirectional, another approach is to demonstrate how actively synchronizing movement to external rhythms (as opposed to passive listening) modifies perception. A growing body of evidence has illustrated that tracking auditory rhythms with finger tapping promotes accurate and robust rhythm perception (Morillon et al., 2014; Dalla Bella et al., 2017; Morillon and Baillet, 2017). However, the evidence is sparse concerning higher rhythmic levels.

A recent study on time perception reported direct comparisons between auditory-motor interaction at different rhythmic levels (Hammerschmidt and Wöllner, 2020). In this study, participants were presented with hierarchically structured musical rhythm patterns. They were instructed to tap their fingers to three different hierarchical levels and subsequently estimated the duration of the stimulus. The results showed that tapping to the highest level led to the shortest time estimation. Moreover, participants who tapped more consistently and accurately reported shorter time estimation. Extending these findings to other aspects of temporal processing will shed light on the auditory-motor interaction as the brain interprets speeches unfolding over time (Iversen and Balasubramaniam, 2016).

### 3.3. Imagining higher-level rhythms: Finger tapping in the absence of external sounds

The finger-tapping task externalizes internal rhythms, with or without an external pacemaker. Constructing imagined rhythm requires the engagement of higher-order brain function in which auditory mental imagery is formed and accurately organized (Lu et al., 2019, 2021). Thus, studying finger-tapping to imagined rhythms could shed light on the top-down mechanisms in encoding and maintaining the hierarchical rhythms.

Motor synchronization to imagined rhythms has been studied using the synchronization-continuation task (e.g., Peters, 1989; Semjen et al., 2000). In this variation of the finger-tapping task, participants first tapped to an external rhythm in the synchronization phase, then reproduced the rhythm without sounds in the continuation phase.

A recent study by Cheng et al. (2022) examined auditory-motor interaction during the imagination of hierarchical rhythms. In each trial, participants first listened to a rhythm containing strong and weak beats (i.e., physical meter condition), then mentally imposed the rhythmic structure onto unaccented sounds (i.e., imagery meter condition). Finally, they reproduced the imagined meter with strong and weak finger taps when the sound stopped. With simultaneous EEG recording, the researchers showed that both auditory and motor neural components tracked the imagined rhythm at the beat and meter rates, with strong bidirectional information flows between auditory and motor systems. In this study, the finger-tapping task mainly served as a verification of correct imagination, thus lacking analyses of neural activities during finger-tapping.

So far, empirical evidence has demonstrated that humans can tap into different levels of rhythm that have differential impacts on perception. These findings indicate finger-tapping tasks as a feasible approach to probing auditory-motor interactions in processing hierarchical rhythms. However, whether this conclusion could be generalized to speech processing requires careful examination, which we addressed in the next section.

## 4. Studying processing of hierarchical rhythms with finger-tapping to speech stimuli

Direct evidence on whether humans can tap into rhythms in speech has been lacking until recently. In their study, Rathcke et al. (2021) showed that finger-tapping is entrained by natural speech. Moreover, participants were more likely to tap into metrically strong syllables, suggesting that tapping is sensitive to the hierarchical rhythm structure (Rathcke et al., 2021).

However, compared with non-speech stimuli, manipulating rhythmic structure in speech is complicated since speech rhythm is shaped by interacting acoustic and linguistic factors (e.g., Fiveash et al., 2021). Therefore, more elaborate designs are needed.

For example, The English language typically placed the stress at the right edge of each syntactic phrase (e.g., “the BOY that HELPED the GIRL got an “A” on the TEST,” stressed syllables were capitalized). In this way, the acoustic rhythms (also known as meter) aligned with the linguistic/syntactical structure (Hilton and Goldwater, 2021). Hilton and Goldwater (2021) took advantage of this feature and examined the effect of meter-syntax alignment on finger-tapping performance. In each trial, participants were presented with a series of auditory tones followed by an auditory sentence. The sentence stimuli contained monosyllable words, with acoustic features (e.g., duration, intensity, and pitch) normalized among syllables. Thus, there were no acoustic cues to meter in these sentences. Meanwhile, the preceding tones were amplitude modulated so that the strong and weak tones set a metrical context that either aligned or misaligned with the syntactical structure of the sentence. Participants tapped their fingers in time with the strong beat of the tones, then continued the pattern on their own during the sentence presentation. At the end of the trial, participants completed a comprehension task. Misaligning meter and syntax caused more comprehension mistakes and, intriguingly, disrupted auditory-motor synchronization: participants showed larger tapping variability during the sentence presentation. This study adds new evidence to the growing literature on the auditory modulation of motor output. More importantly, it provides behavioral evidence for the hypothesis that auditory-motor interaction optimizes speech comprehension, which may explain our natural tendency to gesture while speaking (Hilton and Goldwater, 2021).

Meanwhile, studies in the linguistic field have demonstrated the facilitating role of auditory-motor interaction in speech perception (Falk and Dalla Bella, 2016; Falk et al., 2017). Researchers composed speech stimuli alternating stressed (i.e., accent) and unstressed syllables, thus forming an acoustic hierarchical structure or meter. Participants were instructed to align (“congruent” condition) or misalign (“incongruent” condition) their finger tapping to the stressed syllables while performing a word change detection task. The results showed that congruent alignment of motor rhythm with the stressed syllables resulted in better detection performance than the incongruent condition and perceptual control conditions without finger tapping (Falk and Dalla Bella, 2016). Further, in the congruent condition, detection performance was predicted by participants’ tapping accuracy to non-verbal cueing stimuli (Falk et al., 2017). These findings were consistent with the notion that motor synchronization contributes to auditory temporal attention (Morillon et al., 2014). Notably, in the studies above, the absolute tapping rates were constant among trials. Whether auditory-motor interaction to different rhythmic levels differs in effects remained unanswered.

## 5. Studying auditory-motor synchronization in rhythm processing with the speech-to-speech synchronization task

Aside from the fruitful findings, controversy remains: is finger-tapping, a non-verbal behavior, effective in revealing auditory-motor synchronization specific to speech? The auditory-motor synchronization phenomenon has been found in other effectors, such as foot, head, and torso movements (Bouwer et al., 2021), with evidence that different effectors synchronized to different levels of rhythmic hierarchy (Burger et al., 2014; Pflug et al., 2019). For example, a recent study demonstrated a discrepancy between the dominant hand and the non-dominant hand: for right-handed participants, faster tapping at a beat level was better with the right hand, and slower tapping at a higher level was better with the left hand (Pflug et al., 2019). Moreover, finger tapping and speech production involve different optimal time scales: while the auditory-motor interaction in speech processing is restricted to around 4.5 Hz (Assaneo and Poeppel, 2018), finger tapping could adapt to a wider range of tempi, with an optimal frequency around 1.5 Hz (Zalta et al., 2020). Therefore, conclusions should be made with caution, considering the possible discrepancies between fingers and speech effectors when studying speech-specific auditory-motor interaction.

The recently developed spontaneous synchronization of speech (also referred to as speech-to-speech synchronization, SSS) task evaluates participants’ ability to synchronize speech production, a verbal behavior, with external speech sounds (Assaneo et al., 2019; Lizcano-Cortés et al., 2022). Thus, the SSS task provides a direct measurement of speech-specific auditory-motor synchronization. In this task, participants synchronize their vocalization to syllable sequences presented at a constant rate (typically at 4.5 Hz) or an accelerating rate (e.g., 4.3 to 4.7 Hz in 0.1 Hz steps). The subject vocalization is recorded, and the phase locking value (PLV) of the reconstructed envelope of the recorded and presented signals is computed to measure the synchronization of speech.

The original (Assaneo et al., 2019) and the follow-up studies (Assaneo et al., 2021; Kern et al., 2021) showed that the SSS task stably classified the general population into two groups: high synchronizers and low synchronizers. Aside from superior speech synchronization, several advantages in speech-related cognitions have been demonstrated for high synchronizers, including better performance when learning pseudo-words (Assaneo et al., 2019; Orpella et al., 2022) and discriminating rate differences beyond the typical optimal range (Kern et al., 2021). High synchronizers also showed stronger motor entrainment of speech perception (Assaneo et al., 2021). In this study, subjects performed a syllable discrimination task after rhythmically producing syllable sequences. In high synchronizers only, the perceptual performance was modulated by the phase of the target syllable, which was determined by the production rhythm. Although fewer studies explore the neural basis underlying the behavioral differences, distinct brain features have been found between high and low synchronizers (Assaneo et al., 2019). Specifically, high synchronizers demonstrated enhanced brain-stimulus synchrony in the frontal area, better synchrony symmetry in the early auditory area, and greater left lateralization in the white matter connection between these two areas.

This drastic dichotomy of low- and high-synchronizers has not been spotted by the vast body of finger-tapping studies. Therefore, the SSS task may be more sensitive to speech-specific rhythm processing. To be noted, by far, the SSS task has only been validated in native English (Assaneo et al., 2019) and German speakers (Assaneo et al., 2021). Further studies are needed to assess the generalization to other language populations.

## 6. Discussion

Auditory-motor interaction in speech processing has been an active field for decades (Hickok et al., 2011; Poeppel and Assaneo, 2020). Emerging evidence from finger-tapping studies suggests that this interaction exists not only at the basic rhythmic level (Rathcke et al., 2021; Kliger Amrani and Zion Golumbic, 2022) but also at higher levels, subject to variations in both acoustic and syntactical structures (Falk and Dalla Bella, 2016; Falk et al., 2017; Hilton and Goldwater, 2021). Moreover, the auditory-motor interaction is bidirectional: the perception of higher-level rhythms affects motor synchronization to external sounds (Chen J. L. et al., 2006; Chen J. L. et al., 2008; Kung et al., 2013; Hilton and Goldwater, 2021); meanwhile, active motor synchronization also affects the perception of the sounds (Falk and Dalla Bella, 2016; Falk et al., 2017; Assaneo et al., 2021). However, direct evidence is still lacking. More studies using speech stimuli are needed, as well as direct comparisons of the auditory-motor interaction mechanisms at the basic and higher rhythmic levels. Moreover, as mentioned earlier, the newly developed SSS task measures speech-specific auditory-motor interaction using vocalization as the targeted movement. To our knowledge, no studies have yet used SSS tasks in the context of multiple rhythmic levels. Inspired by finger-tapping studies, foreseen future directions include manipulating the rhythmic structures of the syllables sequence and requiring vocalization to a specific level of the hierarchical structure.

## Author contributions

LL and LxL conceived the focus of the review, reviewed the literature, and finalized the manuscript. Both authors approved the final version of the manuscript.
